# Emerging Role of Cellular Prion Protein in the Maintenance and Expansion of Glioma Stem Cells †

**DOI:** 10.3390/cells8111458

**Published:** 2019-11-18

**Authors:** Stefano Thellung, Alessandro Corsaro, Alessia G. Bosio, Martina Zambito, Federica Barbieri, Michele Mazzanti, Tullio Florio

**Affiliations:** 1 Sezione di Farmacologia, Dipartimento di Medicina Interna & Centro di Eccellenza per la Ricerca Biomedica (CEBR), Università di Genova, 16132 Genova, Italy; stefano.thellung@unige.it (S.T.); ale.corsaro@unige.it (A.C.); alegbosio@gmail.com (A.G.B.); zambito.martina@yahoo.com (M.Z.); federica.barbieri@unige.it (F.B.); 2 Dipartimento di Bioscienze, Università di Milano, 20133 Milano, Italy; 3 IRCCS Ospedale Policlinico San Martino, 16132 Genova, Italy

**Keywords:** cellular prion protein, glioma, cancer stem cells, intracellular signaling

## Abstract

Cellular prion protein (PrP^C^) is a membrane-anchored glycoprotein representing the physiological counterpart of PrP scrapie (PrP^Sc^), which plays a pathogenetic role in prion diseases. Relatively little information is however available about physiological role of PrP^C^. Although PrP^C^ ablation in mice does not induce lethal phenotypes, impairment of neuronal and bone marrow plasticity was reported in embryos and adult animals. In neurons, PrP^C^ stimulates neurite growth, prevents oxidative stress-dependent cell death, and favors antiapoptotic signaling. However, PrP^C^ activity is not restricted to post-mitotic neurons, but promotes cell proliferation and migration during embryogenesis and tissue regeneration in adult. PrP^C^ acts as scaffold to stabilize the binding between different membrane receptors, growth factors, and basement proteins, contributing to tumorigenesis. Indeed, ablation of PrP^C^ expression reduces cancer cell proliferation and migration and restores cell sensitivity to chemotherapy. Conversely, PrP^C^ overexpression in cancer stem cells (CSCs) from different tumors, including gliomas—the most malignant brain tumors—is predictive for poor prognosis, and correlates with relapses. The mechanisms of the PrP^C^ role in tumorigenesis and its molecular partners in this activity are the topic of the present review, with a particular focus on PrP^C^ contribution to glioma CSCs multipotency, invasiveness, and tumorigenicity.

## 1. Introduction

The discovery of molecules with pleiotropic functions in heterogeneous pathophysiologic conditions, is a common event in cell biology. A relevant example of such molecules is the cellular prion protein (PrP^C^), the physiological counterpart of the pathogenic prion protein scrapie (PrP^Sc^). Its name was introduced by Stanley B. Prusiner to indicate the proteinaceous etiologic agent of Scrapie, an endemic and infective neurodegenerative disorder of sheep, representing the prototype of prion diseases, also known as transmissible spongiform encephalopathies (TSEs) [1]. Until a few years ago, PrP^C^, whose precise physiological role is still debated, was almost exclusively associated with the development of TSEs after its tridimensional conformation alteration [2,3,4]. However, recently a possible key role of PrP^C^ in tissue plasticity, embryogenesis, and cancer development is also emerging [5]. PrP^C^ plays a central role in the development of TSEs, being expression in neurons, immunocompetent cells, and peripheral organs, crucial for the neuro-invasion by the infective PrP^Sc^ counterpart, and for the consequent neuronal death. In the absence of PrP^C^, cells become resistant to prion infection, brain propagation, and neurodegeneration [6,7,8]. Several potential roles for PrP^C^ in adult central and peripheral nervous systems (CNS and PNS) were proposed. For example, as far as PrP^C^ homeostatic role in PNS, its axonal expression was reported to be required to peripheral myelin maintenance [9], but a definitive role has yet to be proved. On the other hand, a required-to-life role for PrP^C^ is still unclear: transgenic mice in which PrP^C^ expression was ablated do not evidence lethal abnormalities in adult life [10,11], although alterations in neural development were reported [12]. Remarkable efforts have been performed to identify differential pattern of tissue expression of PrP^C^ in mammals, evidencing the highest expression within CNS and PNS, followed by lymphoid organs, bone marrow (BM) hematopoietic cells, and circulating monocytes [13,14,15,16,17,18]. PrP^C^ expression increases along with postnatal growth, but can be identified, at lower level, also in embryonic nerve tissue, particularly in the late stages of pre-natal development [19,20,21]. Analogously, PrP^C^ is expressed in pluripotent cells, including neuron and glial precursors, hematopoietic and mesenchymal progenitors, both in embryos and in adults, suggesting a potential role of such protein in driving ontogenesis and maintenance of tissue homeostasis [12,22,23]. Importantly, the identification of PrP^C^ expression in stem cells could be at the basis of the observation that the absence of PrP^C^ induces some abnormalities during the brain development as well as in the self-renewal capability of hematopoietic stem cells [24,25,26,27,28,29].

Given the peculiar topology of PrP^C^ and the very high levels of expression with the CNS, it has long been hypothesized that it could represent an adaptor molecule for putative neuroprotective factors. Indeed, a growing number of extracellular ligands and extracellular matrix (ECM) proteins are recently known to form multimers in which PrP^C^ acts as scaffold [30].

Finally, at the beginning of the twenty-first century, PrP^C^ also became a relevant player in the oncology field. In fact, evidence was provided about a relevant role of PrP^C^ in tumorigenesis, cancer progression, acquisition of multidrug resistance (MDR), and metastatic propagation [31,32,33].

Gliomas are malignant astrocytic brain tumors that, according to the World Health Organization (WHO), are classified as grade I (pilocytic astrocytoma), grade II (diffuse astrocytoma/oligodendroglioma), grade III (anaplastic astrocytoma/oligodendroglioma), and grade IV (glioblastoma) [34]. Glioblastoma (GBM) is the most aggressive primary brain tumor with a poor prognosis even after multimodal therapeutic approaches [35]. In fact, GBM rapidly relapses after surgical resection, and is able to resist radio- and chemo-therapies. Several hypotheses, not reciprocally exclusive, have been proposed to determine which cell population gives origin to gliomas [36]. It was reported that malignant transformation can occurs even from mature astrocytes, and neurons, which undergo dedifferentiation [37]. However, in most cases, gliomas might arise from tumor transformation of neural stem cells (NSCs) or committed neural progenitors, along their differentiation path into the subventricular zone [38,39]. In fact, as observed in many other solid tumors, a major cause of GBM development, aggressiveness and relapse is the presence, within the tumor mass, of multipotent cells from which more differentiated cells origin to form the bulk of tumor mass. These poorly differentiated cells grow into specific stem cell microenvironments called tumor niches, which contain heterogeneous cell populations including, beside tumor-promoting stem-like cells, ependymal and endothelial cells, astrocytes, and immune system cells. These tumorigenic cell subpopulations have been named glioblastoma stem cells (GSCs), because somehow represent the pathogenic counterpart of normal neural stem cells (NSCs) [40,41]. As most of the normal stem cells, GSCs display low proliferation rate, self-renewal capability, high activity of DNA-repair machinery and drug extrusion pumps, allowing them to survive to the toxicity of most conventional chemotherapies and provide, upon differentiation, a continuous cellular supply to the tumor mass (see [42] for a specific review). Being GSCs the major cause of tumor relapse and pharmacologic resistance, increasing efforts are currently addressed to identify the cellular determinants that account for their self-renewal capacity.

Interestingly, GSCs were reported to be high dependent on PrP^C^ to:

(i) maintain tumor-initiating activity,
(ii) sustain proliferation and invasiveness,
(iii) acquire multidrug resistance, and
(iv) preserve multipotency and ability to differentiate in non-tumorigenic glioma cells [43,44,45,46]. Finally, as observed in normal tissues, in CSCs PrP^C^ seems to act as receptor or scaffold protein for several extracellular signals dealing with maintenance of self-renewal, adherence, invasiveness, and migration of cells [47].

This review aims to collect and critically analyze the most recent discoveries about the role of PrP^C^ in cancer development and progression, particularly focusing on gliomas and GSCs, and to analyze the possible role of PrP^C^ as a target candidate for novel therapeutic approaches.

## 2. The Cellular Prion Protein

PrP^C^, encoded by the *PRNP* gene, is an extracellular syaloglycoprotein, highly enriched in neurons, that is tethered to the outer leaflet of plasma membrane by a glycosylphosphatidyl-inositol (GPI) anchor [48]. It is structured by an α-helix rich C-terminus, and an unstructured N-terminus tail. In TSEs, PrP^C^ undergoes a structural alteration generating a pathogenic isoform (PrP^Sc^) in which a significant part of the unstructured tail is converted in β-structures [1,49]. This alteration allows PrP^Sc^ to become protease-insensitive, forming intra- and extracellular aggregates responsible of neuronal death. PrP^Sc^ generation is not limited to Scrapie, but is the pathogenic mechanism of all fatal, albeit rare, human prion diseases including Kuru, fatal familiar insomnia, Gerstmann–Straussler–Sheinker, and Creutzfeldt Jacob diseases. These forms have sporadic, inherited, and infectious etiologies in which PrP^C^ either spontaneously converts into PrP^Sc^ form, bear conversion-favoring mutations, or bind to exogenous PrP^Sc^ which acts as a template, respectively [1]. The peculiarity of TSEs is their infective behavior, since PrP^Sc^ can interact with newly synthesized PrP^C^ causing its conversion into the pathological isoform, favoring the spreading of the neurodegenerative lesions. A very recent and intriguing theory proposes that similar pathogenic activity induced by protein misfolding occurs independently from the specific protein involved, in TSEs as well as in other more common and fatal neurodegenerative disorders of the central nervous system including Alzheimer’s, Parkinson’s, and Huntington’s diseases and amyotrophic lateral sclerosis [50,51,52,53,54]. In this context, PrP^C^ was proposed to represent the cellular receptor for Aβ and tau in Alzheimer disease, and α-synuclein in Parkinson disease, being these interactions required for the different misfolded protein neuronal internalization and neurotoxicity [55,56,57,58]. Moreover, the biological activity of oligomers from the different misfolded proteins responsible of all these neurodegenerative diseases, was evaluated using different disease models in vitro and reported to activate similar proapoptotic and gliotrophic pathways [59,60,61,62,63,64]. In particular, data using purified PrP^Sc^ or amyloidogenic PrP^Sc^-mimicking peptide models demonstrated the activation of p38 MAP kinase, excitotoxicity via NMDA receptors and dysregulation of Ca^+2^ homeostasis or autophagy to be the main neurotoxic activity on neurons, while the same treatments caused activation of astrocytes and microglia leading to proliferation via ERK1/2 MAP kinase and release of cytokines, chemokines, prostaglandins and nitric oxide [62,65,66,67,68,69,70,71,72,73,74]. In another experimental setting, ERK1/2 MAP kinase activity, relocated in the cytosol, was shown to favor prion replication, while JNK activity counteracted the formation of prions [75,76].

Given its widespread expression among mammals, it is reasonable that PrP^C^ plays a significant role in brain and other organs functioning that extends beyond sensitivity to prion illness, driving critical processes for the physiology of the nervous and immunity systems. Although PrP^C^ ablation does not induce lethal phenotypes, important evidence showed that nervous tissue development during embryogenesis, as well as the maintenance of hematopoietic and mesenchymal pluripotent cells in adult mammals, requires the presence of PrP^C^ on the cell surface [9,10,11,12,22,28,77].

## 3. Physiology of PrP^C^ in the Development and Homeostasis of Normal Tissues

Mammalian PrP^C^ is mostly expressed in the CNS where it becomes detectable at late stages of embryonal development and strongly increases shortly after birth, though maintaining a marked heterogeneity among different brain areas [19], under the control of nerve growth factor activity [78]. Noteworthy, PrP^C^ mRNA is detectable, although at lower levels, also along peripheral nerves and ganglia and in the sensory neurons as gut plexus, olfactory membrane and retina [19]. Beyond the nervous system, the expression of PrP^C^ is also detectable in adult bone marrow, lymphoid organs, heart, skeletal muscles, and lung [79,80]. Altogether, these data strongly indicated that neuronal and hematopoietic systems may particularly rely on PrP^C^ for their development, survival, and homeostasis.

### 3.1. Role of PrP^C^ in the Development of the Nervous System

**Figure 1:**
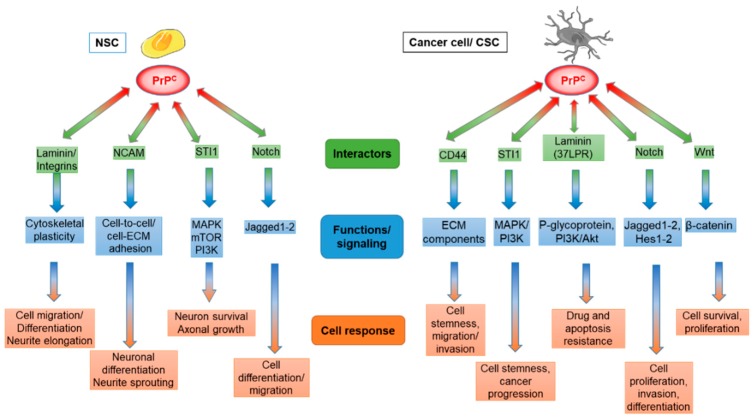
Comparison of cellular prion protein (PrP^C^) engagement to multiple pathways in neural stem cells (NSCs), and cancer/cancer stem cells (CSCs). Partially overlapping interacting proteins between NSCs and CSCs were reported, although the interaction can determine different cell responses (orange in the figure). Abbreviations: NCAM: neural cell adhesion molecule; STI1: stress-inducible protein 1; 37LPR: 37-kDa laminin receptor; ECM: extracellular matrix; MAPK: mitogen-activated protein kinase; PI3K: phosphatidylinositol-3-Kinase; mTOR: mammalian target of rapamycin; Wnt: Wingless-related integration site family; Akt: Protein Kinase B; Hes1–2: hairy and enhancer of split 1–2.

During the embryonic development of neural tube, and in the limited process of adult neurogenesis, pluripotent stem cells and neural precursors differentiate into neuronal and glial lineages [19,81,82,83]. In light of the potential role of PrP^C^ as a scaffold that engages different soluble and/or membrane factors, stabilizing ligand-receptor links [84,85,86], it was proposed that PrP^C^ may work either as a receptor or an adaptor that accompanies embryonic cells through neural differentiation and migration. The enrichment of PrP^C^ into lipid rafts and the growing number of soluble ligands or plasma membrane and ECM molecules able to interact with PrP^C^ led to the hypothesis that this protein might be involved in cell adhesion to other cells or ECM. A growing number of soluble ligands or plasma membrane and ECM molecules have been studied for their capability to interact with PrP^C^ [87] (Figure 1).

Among the different actors controlling cell shape and migration during embryogenesis and neuron regeneration in adults, particular relevance is held by adhesion proteins [88]. Laminins are heterotrimeric proteins expressed on basement membrane which, interacting with members of the integrin family, control the plasticity of cytoskeleton proteins [88,89]. In neurons, this interaction plays fundamental importance in embryonal development of neural system while, in adults, it models cell shape, migration, and neurite expansion or regeneration. The laminin-integrin system recognizes PrP^C^ as a cofactor that can act either through direct binding to laminin or as a scaffold that regulates integrin signaling [90,91]. PrP^C^ also interacts with the non-integrin 37kDa laminin receptor precursor (37LRP) on the cell surface [90,92,93,94,95,96]. Both laminin-PrP^C^ and 37LRP-PrP^C^ binding are specific and recognize the same domain of PrP^C^. In the absence of PrP^C^ expression, the proper interactions between basement laminin and cell membrane are impaired, resulting in abnormalities in cell differentiation, particularly affecting neuritogenesis and axonal growth [90,91,92].

Another plasma membrane protein involved in neural differentiation and plasticity is the neural cell adhesion molecule (NCAM). NCAM is expressed by neurons and glial cells allowing their adhesion to ECM [97]. Prusiner’s group reported, through the isolation of PrP^C^-containing lipid rafts, the presence of high molecular mass protein complexes in which the major component is NCAM [98]. Following studies confirmed that NCAM and PrP^C^ coexist within lipid rafts establishing a specific binding [99,100]. It was proposed that NCAM recruitment by PrP^C^ serves as a signal to promote neuronal precursor differentiation [101,102], neurite sprouting, and elongation through the activation of Fyn-dependent pathways [98,103].

Beyond adhesion molecules, transmembrane and secreted proteins are recently recognized to play a role in nervous system plasticity. Among these, stress induced protein 1 (STI1) and Notch complex are emerging as PrP^C^ interactors. Stress-inducible protein 1 (STI1), also known as Hsp70-Hsp90 organizing protein (HOP), acts a co-chaperonin involved in protein folding. STI1 is mainly expressed as transmembrane protein in neurons, although its secretion from glial cells has also been described. Importantly, this protein displays a specific amino acidic domain specifically recognized by PrP^C^ [104], and their interaction supports neuronal survival and axonal growth [105]. In particular, upon PrP^C^ binding STI1 is retained on cell surface to sustain neuritogenesis and cell survival through the activation of mitogen-activated protein kinase (MAPK) and protein kinase A (PKA), respectively [106,107]. Moreover, PrP^C^/STI1 binding sustains mTOR-dependent protein synthesis through the maintenance of the PI3 kinase-dependent mTOR phosphorylation [107,108] Importantly, NSC self-renewal is also dependent on PrP^C^/STI1 interaction, since neural progenitors from PrP^C^ null mice, or in which PrP^C^ or STI1 were blocked by antibodies, are less able to ensure self-renewal in vitro even in the presence of exogenously added STI1 [109].

Notch1 is a transmembrane protein that contributes to maintain the stemness of neuronal progenitors and to drive the migration of neuronal and glial progenitor cells in embryonal and postnatal brain [110,111]. It has been demonstrated that PrP^C^ depletion in neuroectodermal cells, neuronal stem cells, and in mice embryos produced a down-regulation of Notch 1 receptor and its cognate ligands, Jagged 1 and 2, and of the expression of target genes including nestin, OLIG2 and N-cadherin, depicting a molecular scenario in which PrP^C^ plays a pivotal role in stemness and self-renewal of NSCs [112].

### 3.2. PrP^C^ Stimulates Hematopoiesis

Early studies on PrP^Sc^ infectivity showed that prion neuroinvasion is preceded by its accumulation in spleen, lymph nodes, thymus, and follicular dendritic cells (FCDs), which represent a relevant site where PrP^Sc^ binds PrP^C^ to induce its conversion into the pathologic conformation [15,113,114]. Remarkably, immunodeficient mice are resistant to PrP^Sc^ passage from periphery to the brain but BM restoration can reintroduce the capacity of PrP^Sc^ to replicate into the mice spleen after intracerebral or peripheral inoculation. These observations clearly indicate the importance of the immune cells in TSE pathogenesis [6,115,116,117,118]. Beyond FDCs, PrP^C^ is expressed in hematopoietic cells including myeloid dendritic cells (DC) and, at a lower level, in circulating blood cells as erythrocytes, platelets, and B and T lymphocytes and monocytes, although still discordant reports were produced on the latter cell populations [13,80,119]. It is hence reasonable to hypothesize a role of PrP^C^ in the immune system functionality and development. The ablation of PrP^C^ in mice produces only minor abnormalities in the mature immune system as alteration in monocytes/neutrophils ratio and in DC-T lymphocytes cross-activation, although it does not cause immunodeficiency [120,121]. However, the lack of PrP^C^ is detrimental under prolonged stressful conditions as can be experimentally induced after serial bone marrow transplantation in irradiated animals. Under these conditions, the presence of PrP^C^ is necessary for the regeneration of hematopoietic cells [28]. Moreover, the expression of PrP^C^ in myeloid progenitors increases after total body irradiation, and post-irradiation recovery of hematopoietic stem cells is hampered in PrP^C^-null mice [122]. These studies indicate that PrP^C^ is highly expressed in BM stem cells of mesenchymal and hematopoietic origin, where, in analogy with NSCs, it sustains their indefinite self-renewal. PrP^C^ expression is inducible by stressful conditions in order to sustain the capacity of BM hematopoietic cells to replenish the pools of circulating mature blood cells.

## 4. PrP^C^ and Cancer Stem Cells in Gliomas and Other Tumors

Metastasization, the capacity of neoplastic cells to detach from a primary tumor mass, diffuse through blood and lymph streams, and form tumor colonies in distant body areas, is one of the main causes of cancer deaths. Metastatic cells often display an enhanced proliferation rate, self-repair capacity, and insensitivity to conventional chemio- and radiotherapy. In CNS malignant solid tumors, such as GBM, fatal outcome is caused by the rapid invasion of brain parenchyma that causes tumor relapse, even after extensive reduction of the mass induced by surgical and chemo-radiotherapy. The current hypothesis to explain cancer’s ability to relapse post-surgery and resist cytotoxic drug treatment, indicates that tumor development and progression is sustained by a minority of cells that combine malignant transformation with stem-like multipotency. These cancer stem cells (CSCs) divide indefinitely and originate non-stem differentiated cells that form the bulk mass of tumors [5,40,41,123]. CSCs theory states that alterations of maturation and self-renewal of organ-specific stem cells could be the main factor at the basis of tumorigenesis. Virtually any kind of tumor, even benign in nature [124] possibly contains CSCs. Notably, CSCs display a high MDR efficiency that, altogether with the low proliferation rate and self-renewal capability, confers resistance to conventional chemotherapy. Since CSCs fuel, upon differentiation, a continuous supply of cells to the tumor mass, it is conceivable that they represent the most notable target for really effective cancer eradicating therapies.

Present in virtually all types of gliomas, CSCs are particularly relevant for the rapid recurrence and dissemination of GBM, whence they have been named GBM stem cells (GSCs) [41,125,126]. GBM is characterized by heterogeneous cell populations comprising GSCs and non-stem cancer cells, intermingled with multiple cell types such immune cells (microglia, peripheral macrophages, leukocytes, CD4+ T cells, and Treg), pericytes, and endothelial cells recruited from non-tumor vasculature or by trans-differentiated GSCs, and includes necrotic areas forming a complex microenvironment [127,128,129]. GSCs are functionally resident in niches, due to the homing activity of the chemokine CXCL12 produced by stromal cells [130], whose microenvironment protects them by external insults, including cytotoxic drugs, and favor their maintenance of stemness. GBM niches have been currently characterized in five different typologies, of which the best characterized is the perinecrotic hypoxic niche, in which the relative scarcity of oxygen sustains GSC stemness, and the perivascular niche in which GSCs contribute to the neovascularization of the tumor mass and form which they migrate to distant brain areas [131,132]. Thus, GBM development proceeds by misleading the same pathways that sustain NSCs in brain sub-ventricular zone [42,132].

The characterization of the cellular mechanisms involved in GSC balance between maintenance of stemness and self-renewal capacity, and the differentiation in non-stem tumor cells, represents an extremely challenging issue with potential therapeutic value, though still largely unmet. Particularly complex is also the definition of those elements that mediates GSCs contact with extracellular ligands or with surrounding cells to modulate their self-renewal or differentiation.

Given its peculiar topology on the cell surface, PrP^C^ may act as a receptor for extracellular ligands and proteins, which interacting with the plasma membrane or basement matrix, transduce cell responses favoring cancer development and progression, including enhancement of protein synthesis, apoptosis blockade, maintenance of multipotency, detachment from extracellular membrane, and acquisition of MDR. One of the first indications concerning the relevance of PrP^C^ in cancer biology dates back to the studies by Fan et al., which demonstrated the overexpression of PrP^C^ in gastric carcinomas and gastric cancer cell line, that was correlated with doxorubicin resistance and a highly invasive behavior in vivo [33,133]. PrP^C^-dependent resistance to doxorubicin was showed to be mediated by the expression of P-glycoprotein, a main effector of MDR, and the increase in the Bcl-2/Bax ratio [31]. Similarly, in colorectal and breast cancers, PrP^C^ expression level was shown to be predictive of resistance to chemotherapy, metastatic behavior and, in general, poorer prognosis [31,44,134,135].

PrP^C^ is expressed at high levels also in glioma tissues [107,136] and cell lines [137,138], and cells from this tumor histotype were reported to be dependent on the presence of PrP^C^ to proliferate and acquire multidrug resistance [139]. The expression of PrP^C^ was also analyzed in patient-derived GSC-enriched cultures that are able to continuously grow in stem cell-permissive, EGF/bFGF-containing medium [43]. Among GSC features, these cultures evidenced the capacity to grow as neurosphere, an in vitro index of self-renewal, and, when intracranially injected into immunodeficient mice, are able to develop tumors which reproduce the characteristics of the original GBM. In this study, several evidences supported the role of PrP^C^ in the tumorigenic activity of GSCs: on one hand PrP^C^ protein levels were directly correlated to the in vitro proliferation rate of these cells, and, on the other, most of GSC-like cellular behaviors were strongly affected by PrP^C^ silencing. Down-regulation of PrP^C^ reduced cell growth, clonogenensis and spherogenesis activities, and the ability to develop tumors in animal models [43]. Phenotypically the loss of GSC-like activity was associated with the down-regulation of stem cell marker expression (e.g., Sox2 and Nanog) in favor of differentiation markers, such as glial fibrillary acidic protein (GFAP) [43]. Similarly, it was also shown that stem-like cells derived from established GBM cell lines (U87, U251) contain higher levels of PrP^C^ than the GBM differentiated cell counterpart [137].

These data suggest that the presence of PrP^C^ is critical to maintain of GSC stemness and that its reduction could represent a strategy to force GSC shift towards more chemotherapy-sensitive and differentiated cancer cells.

Another major reason for GBM relapse after adjuvant chemotherapy is the selection, induced by the pharmacological treatment, of GSCs with increased DNA-repairing and antiapoptotic capacities. For example, this phenomenon was observed after treatment with the alkylating agent temozolomide (TMZ), the most commonly used cytotoxic drug for GBM [140,141]. It was observed that, after TMZ treatment of recurrent GBM samples, PrP^C^ expression is increased, possibly contributing to the acquisition of resistance through the binding and inhibition of nuclear translocation of the transcriptional receptor prostate apoptosis response-4 (Par-4) [45], which normally favors apoptosis via the inhibition of the anti-apoptotic protein Bcl2 [142]. Importantly, the down-regulation of PrP^C^ restores Par-4 activity and GBM sensitivity to TMZ [45].

Acknowledging the ability of PrP^C^ to interact with several NSC receptors involved in the control of self-renewal and differentiation, the role of PrP^C^ interaction with stemness-related ligands in GSCs proliferation, invasiveness, and drug resistance has become a major topic of research and is currently under intense investigation. Indeed, a growing number of interactors has been recently identified and proposed to promote GBM malignancy through their co-operation with PrP^C^, also suggesting that PrP^C^ itself could become a relevant target for therapies specifically directed against the CSC subpopulation [47] (Figure 1). Some of the most extensively studied PrP^C^-interacting molecules in cancer cells and, in particular, in CSCs are discussed below.

### 4.1. PrP^C^ and CD44

CD44 is a transmembrane glycoprotein originally described in lymphocytes [143], that functions as adhesion protein to the ECM components hyaluronic acid, fibronectin, and laminin. Although expressed in a wide variety of normal cells, CD44 is overexpressed in cancer cells, including CSCs, in which favors cell survival via hyaluronic acid binding and allowing distant tissue homing after metastasization [144]. In GBM, CD44 is highly expressed in GSCs lying into niches, and it is regarded as a marker for a poor prognosis [145,146,147]. A definite role of CD44 in sustaining GSC activity is still to be determined and it has been proposed that it may vary according to the location of CD44-positive GSCs within the tumor mass: CD44 is involved in the control of stemness in the cells present in hypoxic niches, while it favors their tissue dissemination if it is predominant in perivascular niche GSCs [148,149,150].

Although no evidence is presently available about a CD44-PrP^C^ direct interaction in GBM, such interaction has been demonstrated in gastric and colorectal cancers [33,44], where it allows the formation of metastasis. Moreover, both CD44 and PrP^C^ are overexpressed in breast cancer cell lines resistant to doxorubicin, and PrP^C^ silencing, disrupting this interaction, allows the recovery of drug sensitivity. Similarly, in breast carcinoma tissues, a direct correlation between the overexpression of both CD44 and PrP^C^ was detected in patients unresponsive to neoadjuvant chemotherapy [31].

### 4.2. PrP^C^ and Stress-Inducible Protein 1

Stress-inducible protein 1 (STI1), is a co-chaperonin, firstly described to stabilize the binding between Hsp 70 and 90. STI1 is mostly resident into the cytoplasm although nuclear, membrane-bound and secreted forms have also been described [151,152]. Pull-down experiments demonstrated that STI1 binds PrP^C^ in a high affinity manner, interacting with PrP^C^ hydrophobic region 113–128 [104]. Furthermore, it has been demonstrated that treatment of retinal neurons with STI1 or synthetic peptides matching its PrP^C^-binding site can prevent anisomycin toxicity in vitro, indicating that STI1 represents a trigger for the anti-apoptotic activity of PrP^C^ [106].

PrP^C^ and STI1 are upregulated in GBM and associated with increased tumor growth and poorer survival of patients [153,154]. PrP^C^-STI1 complex has been reported to occur in GBM and, more in general, the relative abundance of PrP^C^ in GSCs has been hypothesized to sustain, through complexing STI1, the maintenance of stemness in astrocytic tumors. In particular, STI1 is overexpressed and secreted by GBM-associated lymphocytes, macrophages, microglia, and astrocytes, and its secretion stimulates the proliferation and migration of tumor cells, but not of normal astrocytes, only if PrP^C^ is present and available for binding [155,156,157,158]. Moreover, PrP^C^ contribution to stemness maintenance in GSC neurospheres, is impaired after STI1 downregulation which inhibits proliferation and self-renewal, both in vitro and in vivo [137]. Of therapeutic relevance, Martin’s group, reported that the formation of PrP^C^/STI1 complexes induces proliferation of GBM cell lines, via the activation of PI3K and ERK1/2, conversely the blockade of this interaction, depleting PrP^C^ or using a STI1-derived peptide mimicking PrP^C^-interacting sequence which prevents the binding, inhibited cell growth [153]. Also using in vivo experimental models, the intratumor delivery of a peptide able disrupt PrP^C^/STI1 interaction, impaired proliferation and promoted apoptosis of GBM cells [153]. Thus, the contemporary presence of both proteins is required to promote GBM growth and this interaction could represent a potential target for innovative therapies.

### 4.3. PrP^C^ and Laminin

The 37LRP and its 67 kDa form (67LR) are both overexpressed in cancers, contributing to tumor dissemination, preventing apoptosis of cancer cells and favoring adhesion to ECM in distant tissues [159,160]. 37LRP is overexpressed in GBM cells than in normal astrocytes, and its activity is necessary to grant these cells with high proliferating and metastatic behavior [160]. Given the role of ECM and laminin in tumor development, it is likely that its activity in promoting tumor cell survival depends on specific receptors or scaffolding proteins present at cell membrane, including PrP^C^ that was shown to specifically bind 37LRP [90,94,96]. Interestingly, also the PrP^C^ paralogue Doppel (Dpl, see below) interacts with 37LRP although the binding sites for Dpl and PrP^C^ were identified in different 37 LRP regions [161]. Importantly, PrP^C^ co-localized with 37LRP in gastric carcinoma tissues and cell lines, to cause P-glycoprotein-dependent and -independent resistance to conventional anticancer agents’ apoptosis [162]. In this model, 37LRP silencing significantly attenuated PrP^C^ induced multi-drug-resistance by sensitizing vincristine-dependent apoptosis through inhibition of Ser/Thr kinase Akt activation. Thus it was proposed that the activation of the PI3K/Akt intracellular signaling may be required to transduce the PrP^C^-37LPR-dependent acquisition of MDR via anti-apoptotic signals [162].

### 4.4. PrP^C^ and Notch

Notch signaling controls multiple developmental processes and adult tissue homeostasis. Notch was shown to mediate cell-to-cell interaction signaling and to control stem cell maintenance, particularly in the CNS [163]. The activation of the four heterodimeric transmembrane receptors (Notch1–4) occurs upon their binding to a high number of specific transmembrane receptors (Delta-like-1-3-4, and Jagged-1-29) expressed by adjacent cells. Upon activation, Notch receptor family release their intracellular domains (NCID) that translocate into the nucleus to regulate the transcription of target genes (e.g., Hairy Enhancer of Split: Hes, p21/Waf1, cyclin D1 and 3, c-Myc, HER2, and Sox2) [164]. Notch1 signaling sustains the progression of some of the most aggressive human malignancies, including leukemia, pancreatic carcinomas, and GBM [164,165,166,167]. In GBM, upregulated Notch1 signaling is often identified in poorly differentiated, high grade tumors and correlates with reduced survival [168,169]. Human GBM tissues and cell lines overexpress Notch1 and its receptors Jagged-1 and Delta-like -1, showing also nuclear localization of NCID and enhanced expression of Notch-induced proteins, Hes1 and Hes2 [165,170]. Independent investigations showed that genetic or pharmacologic downregulation of Notch signaling in GSC primary cultures and cell lines promote antiproliferative and pro-apoptotic activity in vitro, impairing self-renewal and tumorigenicity [165,168,170,171,172,173,174]. Importantly, Notch1 modulation surveils the balance between self-renewal and differentiation of GSCs with the latter, enhanced by Notch1 inhibition [173,174]. In pancreatic carcinomas co-expression of PrP^C^ and Notch1 correlates with poor survival. Moreover, Notch1 binding to PrP^C^, forming a complex with filamin A (FLNA), is necessary to sustain the proliferative and invasive phenotype of pancreatic carcinoma cell lines. Accordingly, PrP^C^ silencing reduced the expression of either Notch1 and Notch target genes, and inhibited cell proliferation and invasiveness [167].

The depletion of PrP^C^ from neuroepithelial cells and transgenic mice [112] or its pathological conversion during mouse Scrapie infection [175] impair Notch activation and altered the early phases of embryonal neurogenesis, indicating that PrP^C^ expression in neural progenitors is required for Notch-dependent maintenance of cell stemness. Starting from the idea that the stem component of GBM arises from glial progenitors and hijacks physiological pathways to ensure its long-term survival, PrP^C^ expression could represent a possible requisite for Notch signaling in tumor tissues. At this regard, the demonstration that PrP^C^ silencing in GSCs hampers stemness and promotes differentiation [43] may suggest the possibility that impairing PrP^C^ signaling or scaffolding activity interferes with Notch activity to induce the differentiation of GSCs in more chemotherapy-sensitive GBM cells. Further studies are warranted to address this issue.

### 4.5. PrP^C^ and Wnt Pathway

Wnt family is composed by several secreted glycoproteins, which directs through the coactivator β-catenin developmental processes during embryogenesis and homeostasis and repair in adult tissues [176]. Among the pathways involved in the regulation of stem cell function, canonical Wnt/β-catenin signaling is pivotal in the control of proliferation, self-renewal, stemness maintenance, and epithelial–mesenchymal transition (EMT) [177,178]. In the absence of Wnt proteins, cytosolic β-catenin is ubiquitinated and degraded by proteasome, keeping the signaling shut down. Conversely, in presence of Wnt, β-catenin accumulates in cytosol, translocates into the nucleus, and activates Wnt target gene transcription [179,180,181,182].

Dysregulation of Wnt signaling has been described in many human cancers, including GBM [183]. In human intestinal cancer cell lines (Caco-2/TC7 and SW480) and normal crypt-like cells, PrP^C^ interacts in cytoplasm and nucleus with the canonical Wnt pathway effectors, β-catenin and transcription factor 7–like 2 (TCF7L2), upregulating their transcription activity [184]. From a functional point of view, PrP^C^ down-regulation, impairing Wnt signaling, inhibits the proliferation of intestinal progenitors [185]. Moreover, PrP^C^ is involved in the growth and formation of intestinal organoids, since they were defective if derived from PrP^C^-knockout mice, in which an altered nuclear β-catenin localization in intestinal crypts was observed, being likely involved in survival and proliferative mechanisms of intestinal progenitors [184]. In virtue of this evidence, PrP^C^ is considered a modulator of Wnt signaling in proliferating intestinal epithelial cells. Moreover, PrP^C^ interaction with Wnt and its pathway effectors was suggested as molecular correlate of oncogenic processes, since they control cell–cell junctions, and Src kinase family activity and are dysregulated during tumorigenesis [185].

## 5. Pro-Prion and Cancer

**Figure 2:**
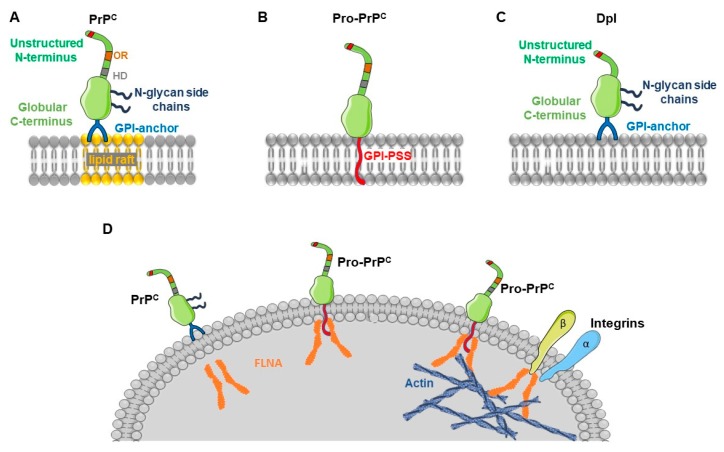
Structural features, cell membrane anchorage and interactions with schematic representation of PrP^C^, Pro-PrP, and Dpl. (**A**) Cellular prion protein (PrP^C^) contains a N-terminal octapeptide repeat domain (OR), a highly conserved hydrophobic signal domain (HD), and a C-terminal hydrophobic region comprising a glycosylphosphatidyl-inositol (GPI) which anchors to the plasma membrane outer face in the lipid raft; (**B**) Pro-prion (Pro-PrP), lacks the N-terminal signal peptide, N-glycan chains and GPI, while it retains the C-terminal GPI anchor peptide signal sequence (GPI-PSS). As a result, Pro-PrP is inserted into the phospholipid bilayer by GPI-PSS rich in hydrophobic amino acids; (**C**) Doppel (Dpl), an N-terminally truncated PrP^C^ protein lacking the octamer repeats. (**D**) Differently form PrP^C^, Pro-PrP interacts with filamin A (FLNA) a cytoplasmic protein involved in actin organization. FLNA acts as cytolinker, which allows the binding of cell surface receptors such as integrins, to F-actin filaments, and forming an actin network responsible for the maintenance of membrane integrity, cell-cell, and cell-matrix interactions.

In normal cells, immature, neo-synthesized PrP^C^ undergoes to endo-proteolytic processing, in endoplasmic reticulum and Golgi before being exposed at plasma membrane. During this process, PrP^C^ loses the 22 amino acid N-terminal signal peptide and a variable C-terminal sequence that is substituted by a glycosylphosphatidyl-inositol (GPI) anchor, and one or two N-linked oligosaccharide residues are potentially added forming mature GPI-anchored PrP^C^ [186]. Intriguingly, a precursor form of PrP^C^, named Pro-PrP is expressed in alternative to the mature form in different cancers, including human pancreatic ductal carcinoma and melanoma cell lines and tissues [187,188,189]. Pro-PrP lacks the N-terminal signal peptide, both sugar moieties and GPI, but retains the C-terminal GPI anchor signal sequence (GPI-PSS). As a result, Pro-PrP is not anchored to the plasma membrane outer face, as mature PrP^C^, but rather it is inserted into the phospholipid bilayer being GPI-PSS domain rich in hydrophobic amino acids (Figure 2). As a potential interactor of Pro-PrP, the actin-binding protein FLNA has been identified in melanoma cells [187]. FLNA connects the cytoskeleton with membrane proteins including integrin β1, promoting cell motility and migration [190]. In particular, Sy et al. [191] identified a specific hydrophobic region of FLNA that recognize GPI-PSS of Pro-PrP (Figure 2). Silencing Pro-PrP in pancreatic carcinoma cell lines, although did not determine a net reduction of FLNA content, modified its intracellular distribution. While in the presence of Pro-PrP, FLNA is remarkably adherent to plasma membrane, in Pro-PrP silenced cells it is diffuse within the cytosol. In both pancreatic cancer and melanoma, the expression of Pro-PrP is associated with a higher propensity to disseminate and a significantly lower mean survival time [191]. It is important to remark that the expression of Pro-PrP was absent in non-neoplastic pancreatic tissues and it was not detectable in all tumors (~40%), but rather it was limited to specimens from most aggressive pancreatic malignancies [191]. Somehow in analogy, FLNA is not expressed in all melanomas indicating that it is not an absolute requirement for the development of these tumors, but its co-expression with Pro-PrP significantly enhance the capacity of melanoma cells to migrate [187]. Altogether, these reports indicate that the unconventional insertion of an immature form of PrP^C^ into the plasma membrane can modify crucial interactions between cytoskeleton and extracellular environment increasing the malignant phenotype of cancer cells. The incomplete processing of PrP^C^ in not associated to any mutation in its coding gene PRNP, rather it is plausible the intervention of mutations in any gene that encodes for proteins involved in the post-translational maturation of PrP^C^ [192].

## 6. Role of the Prion-Like Protein Doppel in Gliomas and Other Cancers

Doppel (Dpl) is a PrP-like protein, encoded by the gene *PRND,* located downstream *PRNP* locus. Structurally, human Dpl and PrP^C^ proteins are similar (Figure 2). Both molecules are glycoproteins exposed on the outside of cell membrane via a GPI anchor, whereas they display a limited amino acid homology (25%). Although Dpl partially overlaps the C-terminal part of PrP^C^, it does not contain the octa-repeats N-terminal region, and the amyloidogenic central sequence 106–126 [193,194]. Moreover, in contrast to PrP^C^, Dpl expression in adults is low in brain and it is mainly confined to testis where it controls spermatogenesis [195,196].

Upregulation of Dpl expression has been identified in an increasing number of cancers including leukemia, lung, colon, and astrocytomas [136,197,198]. Comincini et al. analyzed and compared the expression of PrP^C^ and Dpl in tissue samples obtained from more than 100 astrocytomas encompassing both low and high grade glioma cell lines, as well as other malignancies (anaplastic meningiomas and gastric adenocarcinomas), using adult and fetal normal cortical tissues as controls [136,199]. Dpl was detectable in fetal normal brain, in nearly all analyzed tumors (86%) and in most astrocytoma cell lines, with maximum levels displayed by GBM, and lowest in adult normal brain. In contrast, PrP^C^ showed a strongest expression in normal brain tissues than in GBM samples. The increase of Dpl expression from low to high grade astrocytic tumor samples, allowed the authors to propose that Dpl might represent an early event in tumorigenesis subsequently associated with tumor grading progression.

Little information is presently available to place ectopic expression Dpl within oncogenic events, but recent evidence indicated that its expression may favor tumor cell growth and migration. Al-Hilal et al. demonstrated the presence of Dpl in endothelial cells in tumor-associated vessels in lung and colon carcinomas, and showed that Dpl interacts with vascular endothelial growth factor receptor 2 (VEGFR2) promoting angiogenesis [197]. Noteworthy, a synthetic glycosaminoglycan that binds Dpl causes antiangiogenic effects downregulating VEGFR2 expression [197]. Moreover, genetic manipulation of Dpl levels in astrocytoma cell lines affects migration ability, showing reduced cell motility induced after Dpl silencing and enhanced migratory capacity when Dpl was overexpresses [200]. In virtue of its restriction to neoplastic tissues, Dpl protein could represent either a potential biomarker or a target for selective anti-cancer therapies in the next future.

Intriguing differences between PrP^C^ and Dpl concern also their intracellular localization in GBM cells. While PrP^C^ displayed a typical expression around Golgi and at plasma membrane, Dpl showed mostly cytoplasmic distribution indicating retention into the endosomal-lysosomal system [201,202]. Such unconventional topology of Dpl is more evident in GBM than in low grade astrocytomas, in which most Dpl is tethered at plasma membrane as in normal adult testis [203]. Cancer-related differences of Dpl expression have been found also in BM from patients affected by acute myeloid leukemia [198]. It is thereafter conceivable that Dpl expression and intracellular processing may represent a novel diagnostic marker for different cancer types and its activity, when defined, a potential target for therapy [199,203].

## 7. Concluding Remarks and Future Perspectives

GBM is still one of the deadliest cancers even after aggressive multimodal therapies, but the discovery of GSCs, a milestone for the comprehension of the mechanisms underlying aggressiveness and resistance to therapies, may pave the way for novel targeted therapies. Thus, the main therapeutic challenge is represented by the eradication of GSC subpopulation, which confers the tumor with aggressive behavior, including high invasive activity, and drug resistance. In this scenario, targeting selective molecular drivers which sustain GSC aggressive phenotype, in combination with standard therapies, might improve cancer patients’ treatment response. PrP^C^ is overexpressed in several cancer types, including GBM, and its content is closely related to tumor progression and poor clinical outcome. Multiple CSC-related features, such as resistance to apoptosis, CD44 expression, FLNA activity, MDR, among others, provide this tumor cell subpopulation with a survival, growth, and migratory advantage. Intriguingly, PrP^C^ represents a connecting factor among key stem-related molecules and functions: it interacts with CD44 fueling MDR phenotype, FLNA to promote cell migration, and also with Wnt and Notch signaling exerting pro-survival and proliferative activity.

It is important to remark that several issues are still to be addressed to definitely ascribe to PrP^C^ a prominent role in CSC malignant behavior. For example, it seems contradictory that post-mitotic neurons have the highest PrP^C^ expression but do not proliferate, while GSCs proliferate only in the presence of this protein. While no explanations were provided on experimental basis, it is possible to speculate that in GSCs, at odd with mature neurons, PrP^C^ is irregularly processed to give origin to Pro-PrP which in turn is responsible of the tumor-like behavior. While this could represent a relevant field of study, to date no data were available in GBM, possibly due to the difficulties to discriminate between the two isoforms, that besides topology show very few differences to be evaluated with standard approaches.

It has also to be understood whether PrP^C^ and Dpl have complementary or independent activities in GSCs, to control of tumor cell proliferation and invasiveness.

Few attempts to translate PrP^C^-targeted strategies in anticancer therapeutic applications have been so far published [47]. Considering that PrP^C^ is highly expressed in adult brain and its physiological role in humans is still largely undefined, it is unlikely that a gene therapy for GBM focused on PrP^C^ downregulation, can be devoid of general unacceptable side effects. Thus it is reasonable that effective and low-risk approaches may pursue the inhibition of PrP^C^ and its partners’ specific interactions or the downstream signal transduction pathways. For example, encouraging preclinical results have been obtained by treating GBM xenograft in mouse brains with a peptide that competes with STI1 for PrP^C^, resulting in an inhibition of GBM cell proliferation, without affecting normal astrocyte functioning [153]. Indeed, such interactions, beyond exerting a physiological control of embryonal development and tissue remodeling might also be considered a molecular signature of CSCs in adult tissues, granting a relative tumor-specific interference with GBM cell activity. In prospective, the characterization at the molecular level of PrP^C^ interactions with its partners in tumors and normal cells or the molecules involved in signal transduction activated after the binding is an absolute requirement to develop therapeutic approaches able to discriminate between normal brain cell and tumor signaling.

In conclusion, aimed to propose a view of integration of PrP^C^-associated functions to the GSC malignant phenotype, this review highlighted the relevance of PrP^C^ in oncology, emphasizing its role in cancer prognosis and particularly in CSC phenotype. Future progress in understanding the function of PrP^C^ (and/or Pro-PrP and Dpl) could represent an effective advancement for the development and implementation of PrP^C^-targeting therapeutic strategies improving cancer patient management.
